# F253. A FIDELITY TOOL FOR THE AUSTRALIAN EARLY PSYCHOSIS SYSTEM

**DOI:** 10.1093/schbul/sby017.784

**Published:** 2018-04-01

**Authors:** Eoin Killackey, Kristi van der EL, Heather Stavely

**Affiliations:** 1 Orygen, The National Centre of Excellence in Youth Mental Health; 2 Orygen, The National Centre of Excellence in Youth Mental Health, The University of Melbourne

## Abstract

**Background:**

In the 2010 Australian Federal Budget funding was committed to establish an early psychosis service system based on the EPPIC model of early intervention for psychosis that was developed in Melbourne, Australia. This system was established by 2016. As part of the development of the system a fidelity scale was developed to measure the adherence to the model of the sites delivering the early psychosis services.

**Methods:**

The EPPIC Model Integrity Tool (EMIT) was developed as part of the national reforms around early psychosis. The EMIT is an 80 item assessment tool that maps onto the 16 core components of the EPPIC model. The tool is used by attending the sites and speaking with staff and young people as well as accessing documents, policies and high level data around client flow through the service. The first two rounds of fidelity assessment were conducted in July/August and October/November 2017

**Results:**

Results of the first two rounds of fidelity testing demonstrate a level of fidelity to the model consistent with expectations of each site in relation to their phase of establishment. Data show that there are some components of the EPPIC model that are better implemented than others. These data will give an initial snapshot of the adherence of the sites to the EPPIC model. Also presented will be the means by which reporting back from the assessments to the sites will facilitate closer adherence to the model.

**Discussion:**

Model fidelity is an increasingly recognised way to ensure that programs based on evidence continue to deliver high quality outcomes, and avoid drifting away from the model. This presentation will demonstrate the outcomes from the first two rounds of application of the EMIT and ways in which fidelity testing can help services to improve their support of young people with early psychosis.

